# Mavacamten for Left Ventricular Outflow Tract Obstruction After Successful Transcatheter Aortic Valve Implantation

**DOI:** 10.1016/j.jaccas.2024.103112

**Published:** 2025-03-05

**Authors:** Ha Hye Jo, So-Min Lim, Dayoung Pack, Tae Oh Kim, Sahmin Lee, Byung Joo Sun, Dae-Hee Kim, Jong-Min Song, Duk-Hyun Kang, Jae-Kwan Song

**Affiliations:** Division of Cardiology, Department of Internal Medicine, Asan Medical Center, University of Ulsan College of Medicine, Seoul, South Korea

**Keywords:** aortic valve, echocardiography, mitral valve

## Abstract

Hemodynamically significant left ventricular outflow tract obstruction is a rare complication of transcatheter aortic valve implantation (TAVI). This study presents an unusual case of a patient who, after a successful TAVI, developed and experienced progressive worsening of severe left ventricular outflow tract obstruction after uneventful TAVI that was effectively relieved using mavacamten.

## Graphical abstract

**Figure:**
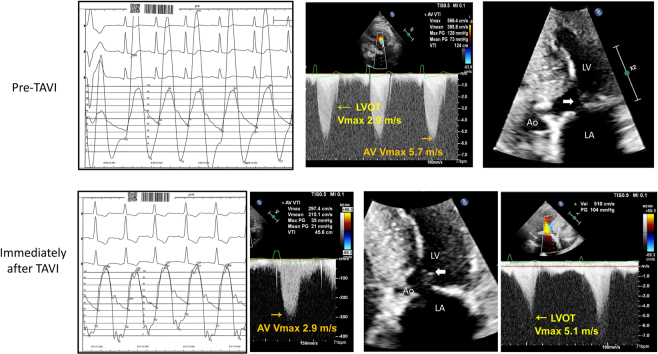

## History of Presentation

An 87-year-old woman was referred for management of progressive dyspnea. At the time of referral, she complained of dyspnea on exertion with NYHA functional class of III. She denied syncope, orthopnea, edema, or palpitation. The patient had a blood pressure of 98/60 mm Hg and a regular pulse (71 beats/min). She had a height of 152 cm and weighed 45 kg (body mass index of 19.5 kg/m^2^). A 4/6 systolic ejection murmur was appreciated on cardiac auscultation.

**Take-Home Messages**

• Significant LVOTO, along with prominent systolic anterior motion of the mitral leaflet, can emerge and worsen following successful TAVI in a patient with valvular AS, potentially leading to progressive heart failure.
• Mavacamten, a currently available myosin inhibitor, demonstrated effectiveness in relieving this hemodynamic compromise, suggesting its potential as a promising therapeutic option for this critical complication.

## Past Medical History

**Figure 1:**
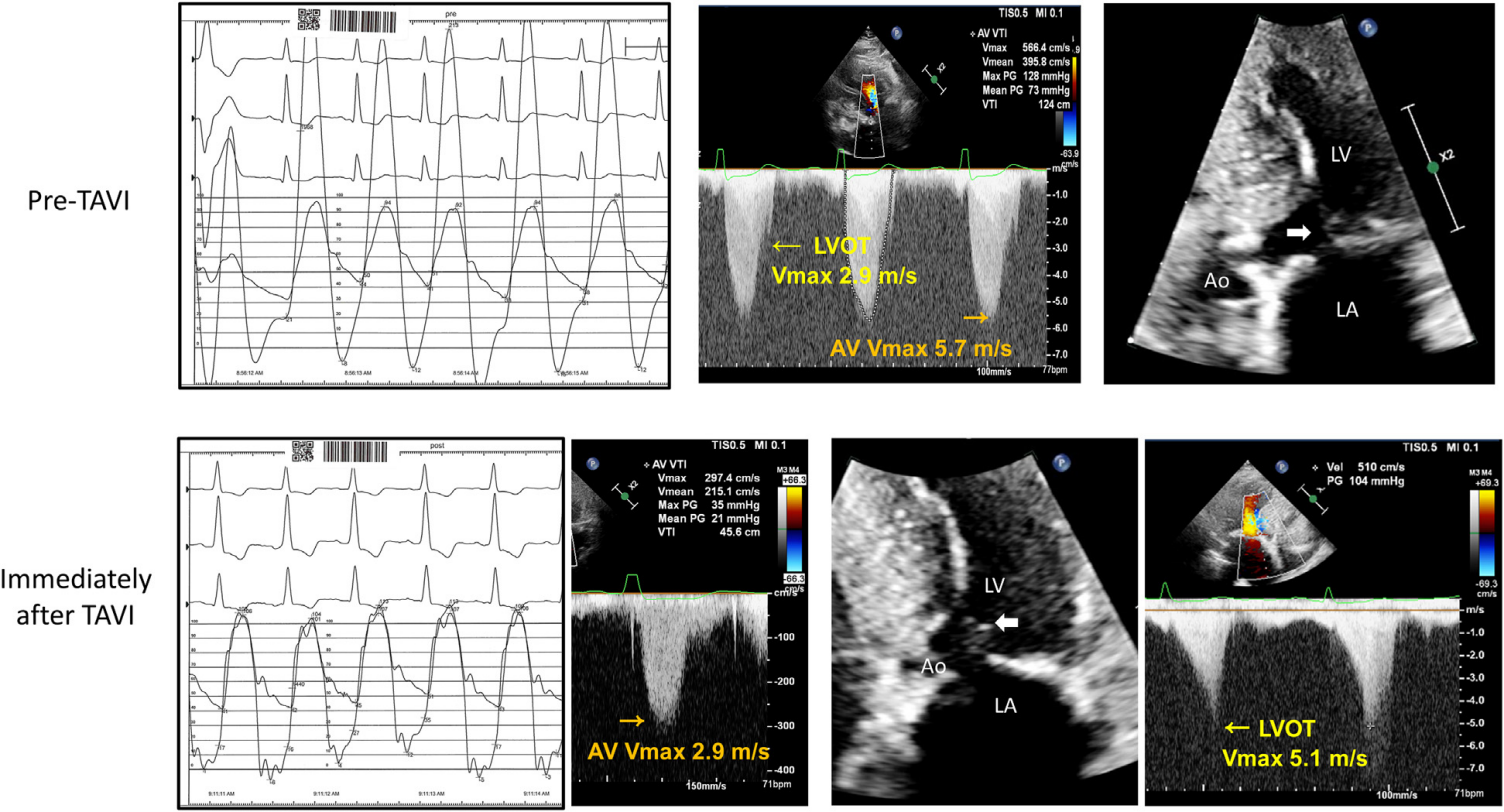
Invasive and Echocardiographic Hemodynamic Assessment Before and After TAVI Before transcatheter aortic valve implantation (TAVI), the systolic anterior motion of the mitral leaflet (SAM, arrow) was not prominent and left ventricular outflow tract (LVOT) flow velocity was not significantly high. Immediately after TAVI, no pressure gradient was present between the left ventricle (LV) and aorta (Ao), but follow-up echocardiography done several days after TAVI showed septal contact of SAM (arrow) resulting in a markedly increased pressure gradient across the LVOT. LA = atrium; Vmax = maximal velocity.

The patient had a long history of hypertension and hyperlipidemia, which were relatively well-controlled with medications. Approximately 1 year before referral, she visited an emergency department due to sudden chest pain and was diagnosed to have acute non–ST-segment elevation myocardial infarction. Coronary stenting was performed at the proximal right coronary artery. At that time, echocardiography revealed severe degenerative aortic stenosis (AS) with a peak aortic velocity of 5.7 m/s and a mean pressure gradient (PG) of 73 mm Hg. Systolic anterior motion of the mitral valve (SAM) and significant left ventricular outflow tract (LVOT) obstruction were not prominently observed (Video 1); however, continuous wave Doppler tracing showed LVOT peak velocity of 2.9 m/s with delayed peak (Figure 1, top), which suggested LVOT obstruction (LVOTO) due to sigmoid septum at baseline and aggravation of LVOTO following afterload reduction after TAVI. The Agatston score of the aortic valve was 2112 and there was no evidence of ATTR amyloidosis on the whole-body bone scan. Transcatheter aortic valve implantation (TAVI) using a 23-mm Edwards SAPIEN valve with 1 mL underfill was successfully completed. The patient was discharged without any complications 2 days after TAVI. However, echocardiography performed immediately post-TAVI showed a mean transaortic PG of 21 mm Hg and the development of SAM and LVOTO with a delayed peak velocity of 5.1 m/s (Figure 1, Video 2). There was no paravalvular leakage. During follow-up, she complained of progressive exertional dyspnea, and the addition of diuretics was not effective (Table 1).

**Table 1:** Summary of Imaging Data, Laboratory Findings, and Medications

	Before TAVI (Baseline)	After TAVI (At Discharge)	12 Months After TAVI	1 Month After Mavacamten
Ao Vmax, m/s	5.7	2.9	3.0	2.5
Mean pressure gradient, mm Hg	73	21	21	13
Aortic valve area, cm^2^	0.53	1.4	1.4	1.4
Degree of SAM	±	++	+++	+
MR severity	+2	+4	+4	+3
Transmitral E/A velocity, cm/s	78/116	154/194	191/91	143/138
Pressure gradient of TR, mm Hg	38	44	64	44
BNP, pg/mL	293	319	716	Not available
Medications, daily dose	Amlodipine 10 mg Valsartan 160 mg Atorvastatin 10 mg	Amlodipine 10 mg Valsartan 160 mg Atorvastatin 10 mg Aspirin 100 mg Clopidogrel 75 mg	Nebivolol 2.5 mg Amlodipine 5 mg Losartan 50 mg Torsemide 5 mg Chlorthalidone 12.5 mg Clopidogrel 75 mg Pitavastatin/Ezetimibe 2/10 mg	Nebivolol 2.5 mg Amlodipine 5 mg Losartan 50 mg Furosemide 20 mg Clopidogrel 75 mg Pitavastatin/Ezetimibe 2/10 mg Mavacamten 5 mg

± = SAM is suspected but not clearly defined; + = anterior mitral leaflet (AML) buckles toward the ventricular septum, but remains 10 mm away from the septum; ++ = AML buckles toward the septum and comes within 10 mm of the septum; +++ = AML buckles toward the septum and contacts the septum; Ao = aortic valve; BNP = B-type natriuretic peptide; MR = mitral regurgitation; SAM = systolic anterior motion of the mitral valve; TAVI = transcatheter aortic valve implantation; TR = tricuspid regurgitation; Vmax = maximal velocity.

## Differential Diagnosis

LVOTO, prosthesis dysfunction including thrombus formation.

## Investigations

**Figure 2:**
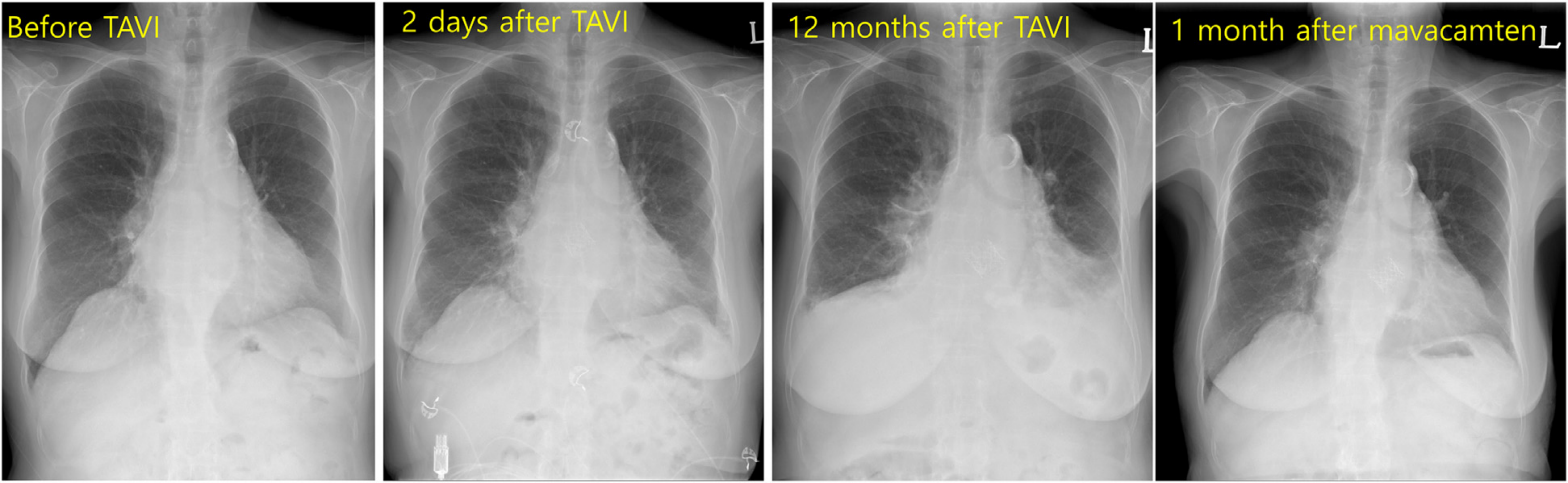
Chest X-Ray Serial chest x-ray showed late development of pulmonary congestion and bilateral pleural effusion despite medical treatment. The addition of mavacamten led to a significant normalization of heart size and the disappearance of bilateral pleural effusion. Abbreviation as in Figure 1.

**Figure 3:**
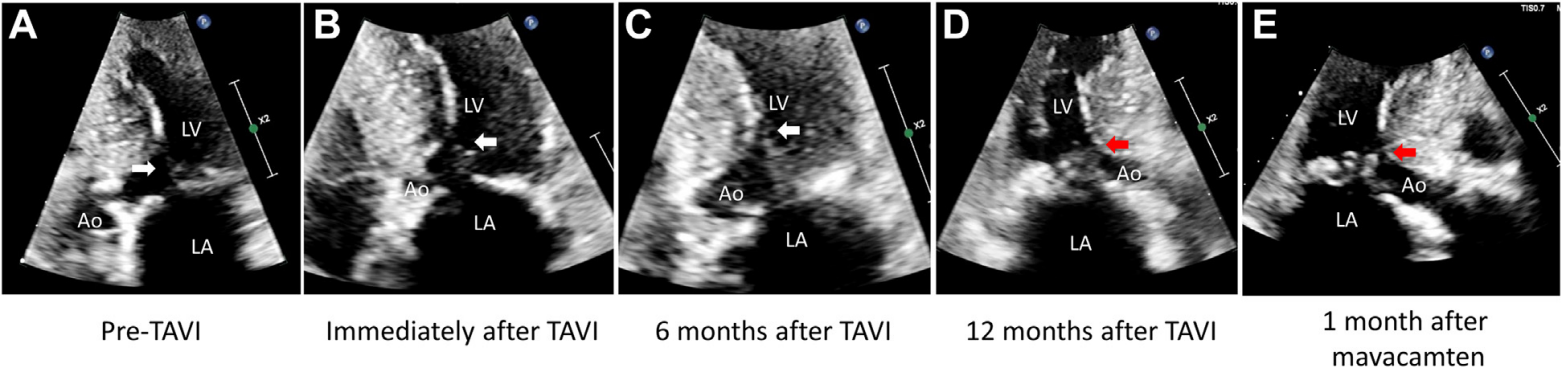
Serial Echocardiographic Images of SAM Before TAVI, SAM was absent (A, white arrow), and immediately after successful TAVI, prominent SAM appeared (B, white arrow), which showed progressive septal contact (C and D, white and red arrows). Mavacamten treatment resulted in less septal contact of SAM (E, red arrow). Abbreviations as in Figure 1.

**Figure 4:**
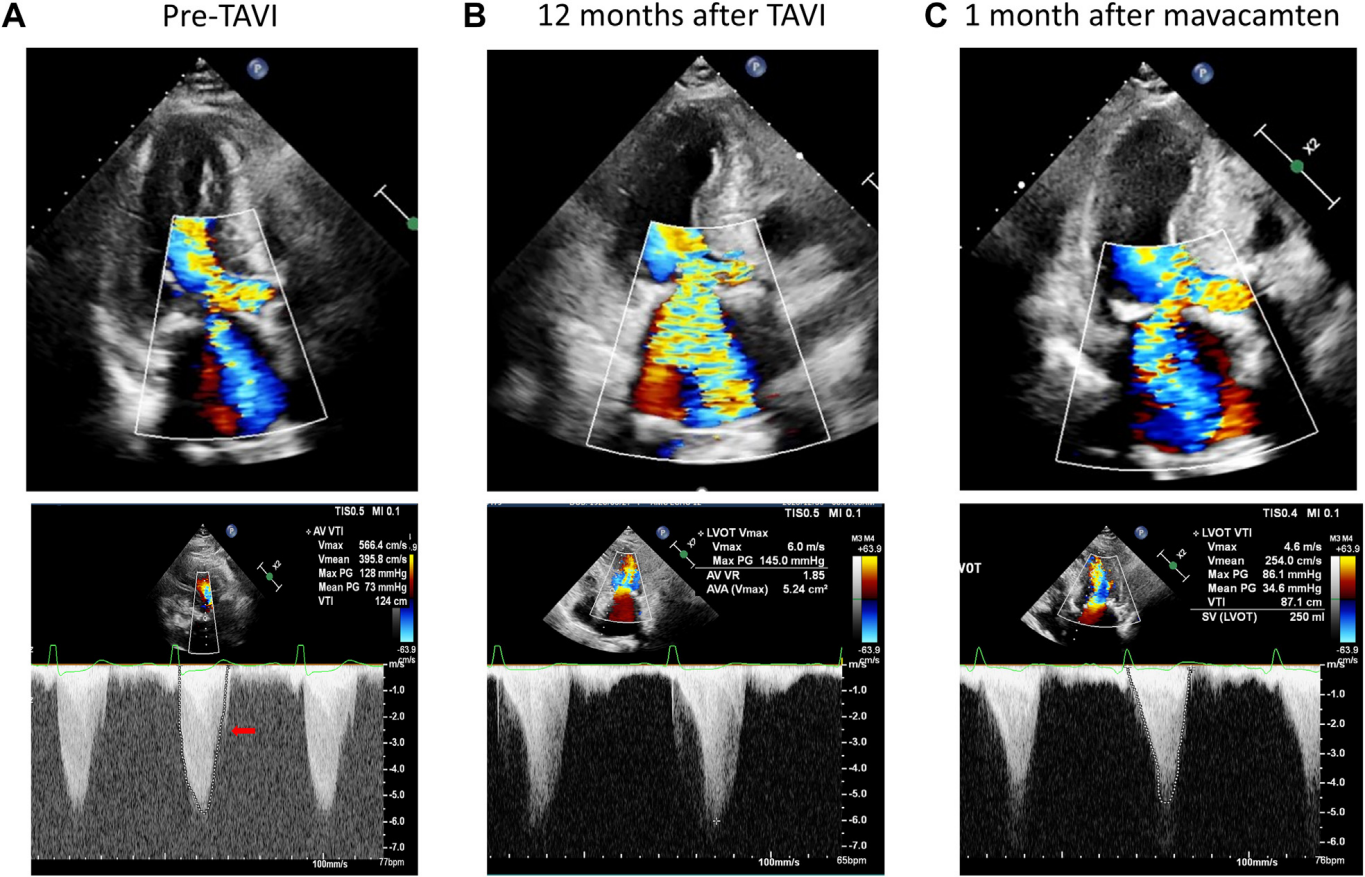
Serial Changes in MR Severity and LVOT Flow Before TAVI, mitral regurgitation (MR) was mild and LVOT velocity was low (A, red arrow). Twelve months after TAVI, severe MR and markedly elevated LVOT velocity were observed (B), which were alleviated with short-term treatment with mavacamten (C). Abbreviations as in Figure 1.

**Figure 5:**
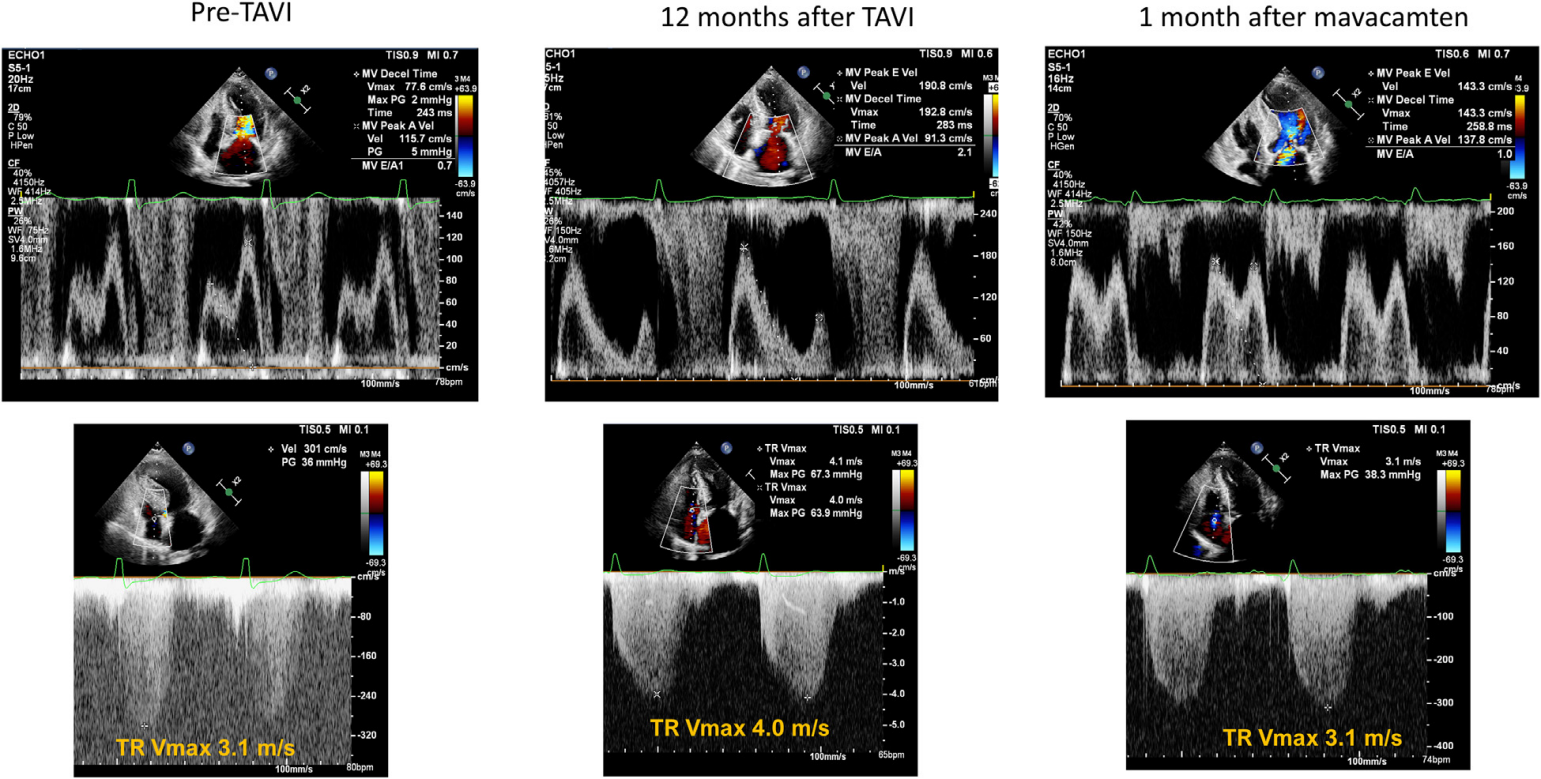
Serial Changes in Doppler Tracing of Mitral Inflow and Tricuspid Regurgitation Jet Before TAVI, an abnormal relaxation pattern of the mitral inflow with mild resting pulmonary hypertension was observed. During follow-up, a restrictive pattern of mitral inflow velocity with significantly elevated pulmonary hypertension was noted, which was reversed with mavacamten treatment. TR = tricuspid regurgitation.

Chest x-ray showed the development of bilateral pleural effusion and increased pulmonary congestion (Figure 2). Follow-up echocardiography revealed a well-functioning aortic prosthesis with a peak transvalvular velocity of 3.0 m/s and a mean PG of 21 mm Hg with the indexed aortic valve area of 0.99 cm^2^/m^2^. Prominent SAM with systolic flow acceleration at the LVOT was persistently observed with the development of septal contact (Figure 3, Video 3). The continuous wave Doppler tracing of LVOT flow revealed a peak velocity of 6 m/s with a delayed peak. Color Doppler flow mapping showed an increased severity of mitral regurgitation (Figure 4): the pulsed Doppler tracing of mitral inflow velocity revealed a dramatic change from an abnormal relaxation patten to a typical restrictive pattern with a marked increase in peak velocity of the tricuspid regurgitation jet from 3.1 to 4.0 m/s, suggestive of aggravated pulmonary hypertension (Figure 5, Table 1).

## Management

Given aggravated symptoms despite optimized traditional medical therapy and the patient’s preference to defer septal reduction therapy (ie, cardiac surgery) or percutaneous transluminal septal myocardial ablation, mavacamten, a currently available myosin inhibitor, was chosen after shared decision-making. Mavacamten was initiated at a dosage of 5 mg daily, and the patient’s condition improved dramatically within 1 month. Chest x-ray showed the disappearance of pleural effusion (Figure 2) and echocardiography showed a decrease in SAM (Figure 2, Video 4), mitral regurgitation severity (Figure 3), peak velocities of LVOT flow (Figure 4), and tricuspid regurgitation jet (Figure 5).

## Discussion

Systolic intracavitary gradients following uneventful surgical aortic valve replacement (SAVR) were reported more than 30 years ago. Hemodynamically, this phenomenon was believed to resemble hypertrophic obstructive cardiomyopathy (HCM), as a typical delayed peak was observed.^1^ The incidence of this phenomenon was later believed to be 14% after SAVR.^2^ Both studies reported the scarcity of the SAM, which suggests different mechanisms including cavity obliteration or midventricular obstruction. A small, hyperdynamic, and asymmetrically hypertrophied ventricle was reported to be a risk factor for the development of abnormal systolic intraventricular flow velocities after SAVR.^2^ Although valvular aortic stenosis is usually associated with a more uniform or symmetric (ie, concentric) distribution of left ventricular hypertrophy in which all segments of the wall show the same or similar thickness, asymmetrical septal hypertrophy (ASH) (a septal-free wall ratio ≥1.3) is reported to be found in approximately 10% of patients with hemodynamically significant valvular AS.^3^ Histologic analysis of left ventricular myocardium may aid in determining whether a given patient with valvular AS has HCM, as the extent of disorganized arrangement of cardiac muscle cells in the ventricular septum or left ventricular free wall is a characteristic morphologic feature of myocardium in patients with HCM. ASH in patients with AS is believed to be an adaptive mechanism to the long-standing pressure overload, and after successful valve replacement, a significant reduction of ASH has been reported.^4^

“Suicide left ventricle” (LV) refers to sudden hemodynamic deterioration after successful intervention of AS, and midventricular obstruction without SAM was initially suggested as the mechanism of this potentially fatal event.^5^, ^6^, ^7^, ^8^ The almost instant alleviation of the transvalvular gradient after TAVI or SAVR exposes the LV to an abrupt significant afterload reduction.^7^ As a result, LV hypercontractility and dynamic intraventricular gradient may develop or worsen, leading to potential resistant circulatory collapse. Prompt recognition of this phenomenon after excluding common causes of shock such as hemorrhage, aortic injury, or coronary artery obstruction is critical to rescue hemodynamic decompensation. Rapid saline loading to increase preload and phenylephrine infusion to increase afterload should be initiated, and all inotropes should be discontinued to decrease LV contractility. Some patients may require full veno-arterial extracorporeal membrane oxygenation support.^6^

Our case has several unique features. First, LVOTO due to prominent SAM, rather than midventricular obstruction or a dynamic intraventricular gradient due to LV hypercontractility, was the main mechanism. In a recent systematic literature review analyzing 25 publications on suicide LV, SAM was present in 76% of cases, and LVOTO, rather than midventricular obstruction, was the most common site of obstruction resulting in acute hemodynamic compromise.^9^ Thus, although the development of midventricular obstruction is more commonly observed after TAVI and LVOTO; this might represent a more frequent but more benign finding. Although a sudden decrease in afterload with the successful intervention of AS (TAVI or SAVR) is expected to contribute to the development of prominent SAM, but it is not clear why SAM was present in patients with valvular AS. There was no evidence that our patient also had HCM, and none of the cases showing SAM after TAVI reported in the literature were reported to have the coexistence of AS and SAM. One notable finding is a potential association between sigmoid septum and development of LVOTO in the aged population.^10^^,^^11^ Further investigation is needed to determine whether mitral leaflet elongation, a key component of the development of SAM, can occur in patients with valvular AS, as ASH accompanies LV pressure overloading.

The second unique feature is that LVOTO can result in the progressive development of heart failure, rather than acute hemodynamic compromise. This patient could be discharged 2 days after TAVI and, at that time, SAM and significant LVOTO were documented. Her condition deteriorated progressively despite the standard medication to decrease inotropy and frank heart failure symptoms developed several months later after successful TAVI. The most unique finding of our case is that medical treatment using a brand-new myosin inhibitor introduced for patients with HCM and LVOTO^12^ was very effective in improving her condition. Many studies have reported notable limitations in traditional methods that were recommended for patients with an intractable clinical course, including invasive interventional procedures such as alcohol septal ablation, right ventricular pacing, or mitral clip.^9^^,^^13^ Our case suggests that the currently available myosin inhibitor can be a promising therapeutic option to improve the clinical condition in patients with valvular AS who developed significant LVOTO due to prominent SAM. Further investigation with cumulative clinical experiences is necessary to confirm the efficacy of this approach in patients with suicide LV with different mechanisms, including midventricular obstruction.

## Conclusions

This is the first documented case of the alleviation of hemodynamically significant LVOTO caused by prominent SAM that became prominent after TAVI in a patient with valvular AS.

## Funding Support and Author Disclosures

The authors have reported that they have no relationships relevant to the contents of this paper to disclose.

## Appendix

Video 1 Before TAVI, SAM or LVOTO was not evident.

Video 1_2 No significant flow acceleration at LVOT on color Doppler flow mapping.

Video 2 Immediately after TAVI, prominent SAM occurred.

Video 3 During follow-up, septal contact of SAM resulted in severe LVOTO.

Video 3_2 Prominent flow acceleration signal at the septal contact of SAM was observed on color Doppler flow mapping.

Video 4 Mavacamten effectively alleviated septal contact of SAM and decreased LVOTO.

Video 4_2 Markedly decreased flow acceleration signal at LVOT with mavacamten medication.
